# Emodepside: the anthelmintic’s mode of action and toxicity

**DOI:** 10.3389/fpara.2024.1508167

**Published:** 2024-12-10

**Authors:** Charity N. Njeshi, Alan P. Robertson, Richard J. Martin

**Affiliations:** Department of Biomedical Science, College of Veterinary Medicine, Iowa State University, Ames, IA, United States

**Keywords:** emodepside, SLO-1K, toxicity, anthelmintic, pharmacokinetics, river blindness, hookworm, *Trichuris*

## Abstract

Nematode parasitic infections continue to be a major health problem for humans and animals. Drug resistance to currently available treatments only worsen the problem. Drug discovery is expensive and time-consuming, making drug repurposing an enticing option. Emodepside, a broad-spectrum anthelmintic, has shown efficacy in the treatment of nematode parasitic infections in cats and dogs. It is now being considered and trialed for the treatment of onchocerciasis, trichuriasis (whipworm), and hookworm infections in humans. Its unique mechanism of action distinguishes it from traditional anthelmintics, positioning it as a promising candidate for combating resistance to other current drugs. Here, we provide a brief review of the available information on emodepside’s pharmacokinetics, safety, and tolerability. We highlight the potential benefits and risks associated with its use, examining key toxicity effects. By exploring the literature, we aim to provide insights into the risks associated with emodepside that may impact its application in veterinary and human medicine. Although emodepside demonstrates a favorable safety profile, continued monitoring of its toxicity is crucial, particularly in vulnerable populations. This mini-review serves as a concise resource for researchers and clinicians interested in anthelmintic therapy.

## Introduction

**Figure 1:**
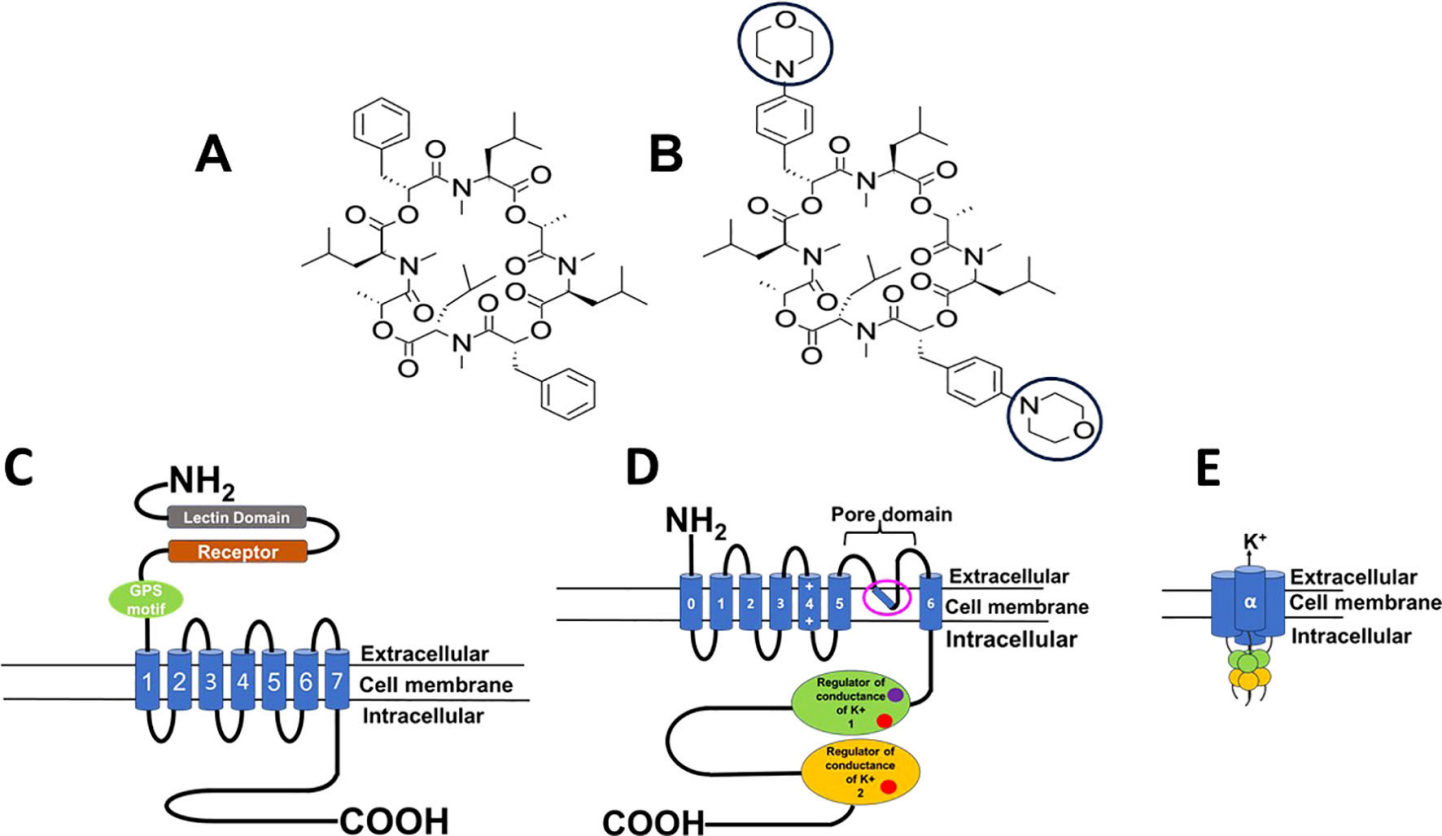
Diagram of structure and protein targets of PF1022A & emodepside. **(A)** Structure of PF1022A, cyclo[(αR)-α-hydroxybenzenepropanoyl-N-methyl-L-leucyl-(2R)-2-hydroxypropanoyl-N-methyl-L-leucyl-(αR)-α-hydroxybenzenepropanoyl-N-methyl-L-leucyl-(2R)-2-hydroxypropanoyl-N-methyl-L-leucyl]. **(B)** Structure of emodepside, cyclo[(αR)-α-hydroxy-4-(4-morpholinyl)benzenepropanoyl-N-methyl-L-leucyl-(2R)-2-hydroxypropanoyl-N-methyl-L-leucyl-(αR)-α-hydroxy-4-(4-morpholinyl)benzenepropanoyl-N-methyl-L-leucyl-(2R)-2-hydroxypropanoyl-N-methyl-L-leucyl]; the morpholino group is circled, adapted from Krücken et al., 2021. Emodepside was discovered by the Japanese pharmaceutical company Astellas and then developed and commercialized by Bayer Animal Health. It is prepared by attaching a morpholine ring on each of two D-phenyllactic acids to PF1022A, a fungus, *Mycelia sterile* of *Camellia japonica*. **(C)** Diagram of the 7-transmembrane structure of the latrophilin receptor. **(D)** Diagram of one of the subunits of the tetrameric SLO-1K receptor; pink open circle, putative emodepside binding site, Raisch et al., 2021; calcium binding sites (red circle) and magnesium binding site (purple circle) **(E)** The tetrameric structure of the α subunit of the SLO-1K channel; Images created in PowerPoint Microsoft.

Emodepside is a semisynthetic compound belonging to the cyclooctadepsipeptide group, known to show anthelmintic activity against larval and adult stages of filarial nematodes (Hübner et al., 2021). It is a member of the *N*-methylated cyclooctadepsipeptides, derived from a naturally occurring compound PF1022A, which was isolated in the early 90s from *Mycelia sterilia*, a fungus that inhabits leaves of *Camellia japonica* (Krücken et al., 2021; Sasaki et al., 1992). Emodepside is synthesized by adding two morpholine rings in para-position of each of the 2 (*R*)-phenyl lactic acids of the parent compound PF1022A (**Figures 1A, B**) (Krücken et al., 2021).

Emodepside was developed as a veterinary anthelmintic and has been found to have broad-spectrum activity against nematode infections of humans (Hübner et al., 2021; Bah et al., 2021). Emodepside is being trialed for the treatment of whipworm, hookworm, and onchocerciasis in humans (Mrimi et al., 2023; Gillon et al., 2021). Adverse reactions though mild and reversible, have been reported and seen to increase with dose (Mrimi et al., 2023; Gillon et al., 2021). The veterinary experience of the use of emodepside in dogs and cats has also drawn attention to some of its toxic effects (Gaens et al., 2019; Walther et al., 2016). This mini-review comments on emodepside’s actions, uses as an anthelmintic and toxicity characteristics of emodepside.

## Spectrum of activity

### Effects on laboratory animal nematode parasites

**Figure 2:**
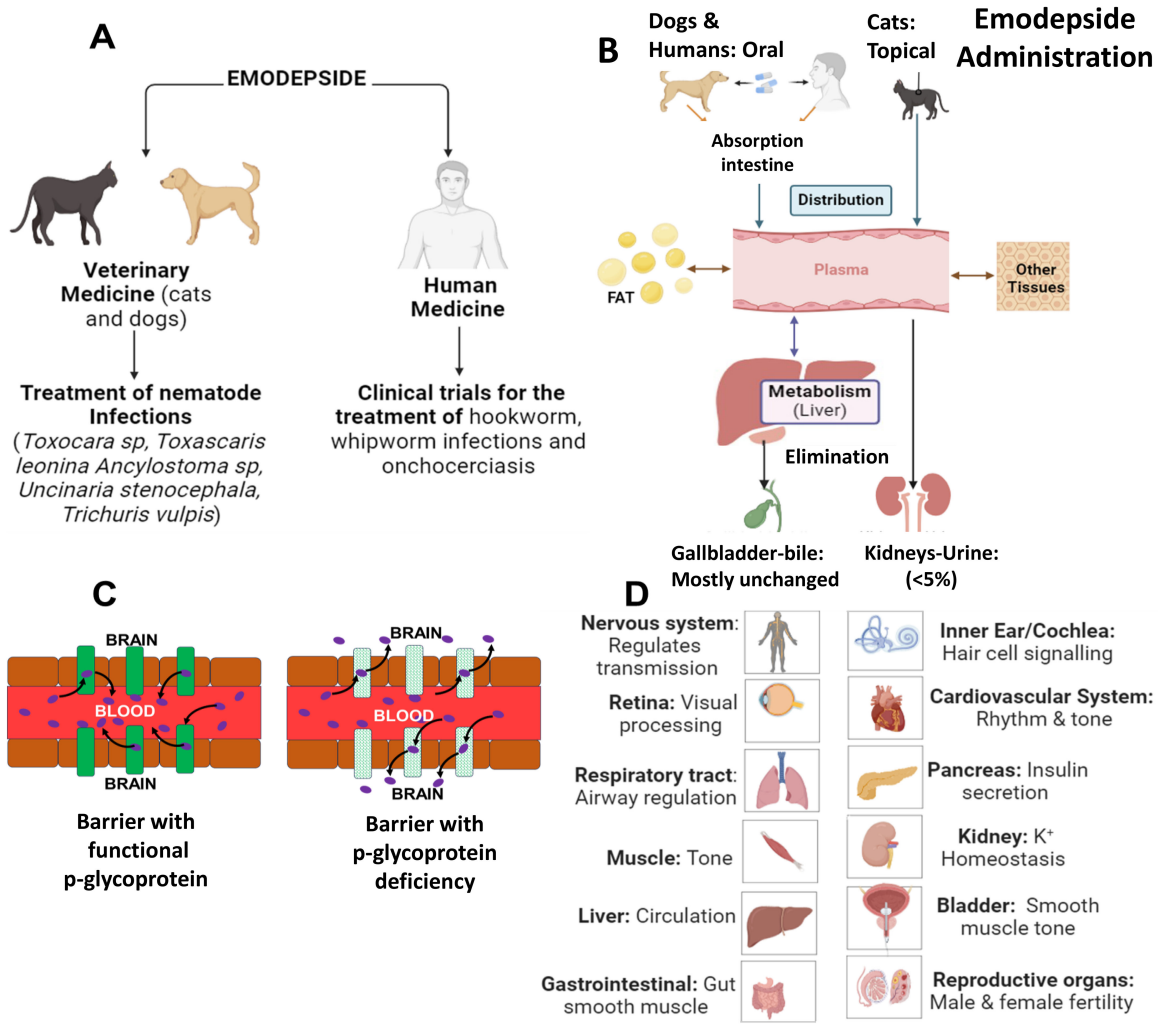
Use of emodepside as an anthelmintic in dogs, cats and applications in humans, pharmacokinetics BK channel target locations. **(A)** Emodepside is effective against a broad spectrum of nematode parasites of cats, dogs, and humans. **(B)** Proposed pharmacokinetics pathways for emodepside. **(C)** Emodepside as a substrate for P-glycoprotein (EMA, 2011). A blood-brain barrier with functional p-glycoprotein (left) prevents emodepside from accumulating in the brain. In contrast, a blood-brain barrier defective for the carrier protein (right) lets emodepside into the brain (purple spheres are emodepside, green rectangles are functional p-glycoprotein, and pattern-filled green rectangles are mutants for p-glycoprotein). **(D)** Wide distribution and functions of BK channels in the human host (Latorre et al., 2017; Peppin et al., 2021; Perry and Sandle, 2009; Delgado-Bermúdez et al., 2024; Henquin et al., 2017; Raisch, 2024; Kunz et al., 2002) *NB:* BK channels are found in more organs, these are examples; Images created by BioRender.com and in PowerPoint Microsoft.

Emodepside has broad-spectrum activity against a wide range of animal nematode soil-transmitted parasites, including those of dogs and cats (**Figure 2A**). *In vitro* studies have also revealed robust activity against larval and adult stages of several nematode species that serve as models for human soil-transmitted helminths and filarial nematodes (Karpstein et al., 2019; Zahner et al., 2001). Sensitivity to emodepside varies between the worm’s life stage and between the different species of parasite (Karpstein et al., 2019).

### Veterinary applications

Emodepside’s spectrum of action extends to nematode parasites of horses, sheep, cattle, chickens, dogs, and cats (Epe and Kaminsky, 2013; Harder et al., 2003). Importantly, emodepside has shown notable activity against nematode parasites resistant to other classes of anthelmintics, such as anthelmintic-resistant populations of *Haemonchus contortus* and *Cooperia oncophora* in sheep and cattle, respectively (Harder et al., 2003; Samson-Himmelstjerna et al., 2005).

Emodepside is marketed for the treatment of parasitic nematode infections in dogs and cats. Emodepside is registered for use in combination with praziquantel (as Profender^®^) for the treatment of hookworm infections, ascarids, and other nematode infections in cats but not dogs in the US, while in Europe, it is approved for use in both cats and dogs (EMA, 2008b; Krücken et al., 2021; Miltsch et al., 2012). This might be due to emodepside’s adulticide activity, which could severely harm heartworm infected dogs in the US, where this disease, in contrast to Europe, is a major threat to dogs. Furthermore, emodepside is recommended by parasitology researchers and the American Animal Hospital Association for off-label use as a last resort for treating multi-anthelmintic drug resistant (MADR) hookworms in dogs. However, its rapid killing for adult heartworms in positive dogs increases the risk of pulmonary embolism and anaphylaxis (Jimenez Castro and Kaplan, 2020; Singler, 2024). Profender is administered orally (as tablets) in dogs and topically (as spot-on) in cats. Emodepside is also part of the spot-on combination with praziquantel and tigolaner (Felpreva^®^) in cats (Blazejak et al., 2023) for treating: roundworms, lungworms, & hookworm (active emodepside); tapeworms (active praziquantel); and fleas, ticks & mites (active tigolaner). Additionally, emodepside is combined with toltrazuril (Procox^®^) for the treatment of nematode parasites (active emodepside) and coccidia (active toltrazuril) in dogs and puppies (EMA, 2011). Furthermore, emodepside is used off-label as a last resort for treating multi-anthelmintic drug-resistant (MADR) hookworms in dogs; however, its rapid killing of heartworms in positive dogs increases the risk of pulmonary embolism and anaphylaxis (Singler, 2024).

Emodepside’s broad spectrum of activity and lack of significant toxicity in animals have prompted the proposal to repurpose and trial it for the treatment of onchocerciasis trichuriasis (whipworm), and hookworm in humans.

### Potential human use: onchocerciasis, hookworm and trichuriasis

#### Onchocerciasis (river blindness)

Emodepside has shown potent activity against both microfilariae and adult *Onchocerca* worms in preclinical and early clinical studies (Bah et al., 2021; Assmus et al., 2022). In the bovine model of onchocerciasis (*O. ochengi*), treatment with emodepside yielded rapid and sustained suppression of microfilariae, with paralysis and eventual death of adult female worms (Bah et al., 2021). This finding is significant because current treatments (like ivermectin) primarily target microfilarial stages, allowing the adult worms to survive, albeit somewhat compromised. Phase I clinical trials in healthy volunteers have reported favorable safety and tolerability profiles with no significant adverse effects observed (Gillon et al., 2021). Building on these promising results, Phase II clinical trials have been planned (Assmus et al., 2022) to assess its efficacy in treating onchocerciasis in endemic regions, and results are awaited.

#### Hookworm

The efficacy of emodepside against *Ancylostoma caninum* (Cima, 2021), encouraged its trial for the treatment of hookworm infections in humans. The drug underwent Phase II trials and demonstrated dose-dependent efficacy (Mrimi et al., 2023). The most common adverse events reported were headache, blurred vision, and dizziness, which increased with higher doses, although they were mild and self-resolving (Mrimi et al., 2023).

#### Trichuriasis

The efficacy of emodepside for treatment against *Trichuris vulpis* infection in dogs (EMA, 2008b) encouraged its trialing in humans. Emodepside has now been evaluated in Phase II clinical trials for the treatment of *Trichuris trichiura* (whipworm) infections in humans (Mrimi et al., 2023). The study reported that 5mg of emodepside per participant produced an 85% cure rate compared to the 17% cure rate in the albendazole control. This observed efficacy suggests that emodepside could become a valuable addition to the limited treatment options for human whipworm infections.

Overall, the available evidence underscores emodepside’s potential as a promising treatment option for onchocerciasis, hookworm, and *Trichuris* infections in humans. However, additional Phase III trials are still required to assess its efficacy and safety under field clinical conditions for the treatment of these neglected tropical diseases.

## Mechanism of action of emodepside

### Latrophilin-like receptors

Early attempts to decipher the mode of action of emodepside (Bay44-4400) using the natural compound (PF1022a) led to the reports that it serves as a ligand for the *Haemonchus contortus* HC110-R protein, which is structurally similar to the mammalian latrophilin G-coupled protein receptor (**Figure 1C**) (Saeger et al., 2001; Willson et al., 2004). Latrophilin receptors are expressed in mammals and other organisms, in various tissues, and abundantly in the nervous system, where they play an important role in neurosecretion for neuronal development and function (Moreno-Salinas et al., 2019; Guest, 2008; Harder et al., 2005; Silva and Ushkaryov, 2010). Emodepside was reported to inhibit pharyngeal pumping and locomotion in *Caenorhabditis elegans* (Amliwala, 2005; Willson et al., 2004). The effects of emodepside on pharyngeal pumping were inhibited by double mutants of *lat-1* and *lat-2*, but the inhibitory effects of emodepside on locomotion were not abolished (Guest et al., 2007). This suggested that emodepside also acted on another receptor, subsequently identified as the SLO-1 K channel (Guest et al., 2007).

### SLO-1K channels

Calcium-activated potassium channels referred to as SLO-1K, also known as BK (“big potassium”), channels are large-conductance potassium ion channels composed of four α subunits that are each seven-transmembrane proteins (**Figure 1D**). These α subunits assemble around the pore to form a tetramer (**Figure 1E**) They are calcium-activated, voltage-sensitive channels that are selective for potassium ions and exist in various isoforms. SLO-1K channels are widely distributed across various tissues of nematodes, playing critical physiological roles in neurotransmitter release, muscle function, and cell excitability (Gessner et al., 2012; Salkoff et al., 2006; Nowicka-Bauer and Szymczak-Cendlak, 2021). Studies have shown that emodepside acts directly on SLO-1K channels, activating them and causing hyperpolarization resulting in paralysis of the worm (Bull, 2008; Buxton et al., 2011; Holden-Dye et al., 2012). Two binding sites have been suggested: one beneath the channel’s selectivity filter, as shown by Cryo-EM experiments (Raisch et al., 2021), and a putative site on the channel’s RCK1 domain based on molecular docking (Kashyap et al., 2019). The effect of emodepside on SLO-1 channels in *Ascaris suum* muscle is enhanced by activating the protein kinase C, and NO pathways (Buxton et al., 2011) and diethylcarbamazine-mediated action on TRP channel (Verma et al., 2020; Kashyap et al., 2022) showing that other signaling pathways can modulate the effect of emodepside on these channels.

We point out here that latrophilin receptors and SLO-1K (BK) channels are present in mammalian hosts. This implies that there is a possibility of observing undesirable effects of emodepside on these receptors in the human and animal hosts of the nematode parasites. Nevertheless, it is possible that its anthelmintic specificity is conferred by other mechanisms unique to the target parasite (Raisch and Raunser, 2023).

## Pharmacokinetics of emodepside

The pharmacokinetics of emodepside have been investigated in rats where rapid absorption and distribution throughout all organs were observed; the highest concentrations were found in the fat following oral administration (EMA, 2008b). A secondary peak has been seen after four days in cats following oral administration, which was suggested to be due to emodepside having a slow redistribution from the fat back into plasma (Page, 2008). Enterohepatic recycling may contribute to a secondary peak that is seen following administration. Compared to cats, dogs show a shorter elimination time due to both faster metabolism and differences in the route of administration (EMA, 2008b).

In the human Phase I clinical trials with oral administration for onchocerciasis, the reported pharmacokinetic profile of emodepside, was rapid absorption, fast distribution to tissues, and an extended half-life of more than 500 hours in the fasting state: in the fed state emodepside was absorbed more slowly (Gillon et al., 2021, 2020). This contrasts with dogs, where there is more rapid absorption in the fed state (EMA, 2008a). A rapid absorption in humans produces a high peak plasma concentration, Cmax, which is connected to emodepside’s activity against microfilariae, while the long terminal half-life is also important for the elimination of the tissue-dwelling macrofilariae (Gillon et al., 2021).

Emodepside is primarily excreted through bile and then eliminated in the feces, with small amounts in urine, indicating minimal metabolism (CTP, 2014; Vercruysse and Claerebout, 2022; Mencke et al., 2023) and safety for patients with renal impairment. The major excretion products of the drug include unchanged emodepside and hydroxylated derivatives (Mencke et al., 2023; EMA, 2008a), suggesting a level of hepatic metabolism. The liver’s role in metabolism and enterohepatic recycling remains to be characterized. The excretion of a significant proportion of unchanged drug suggests its stability and a reduced risk of forming harmful metabolites and toxicity.

The lipid-soluble nature of emodepside decreases its bioavailability following oral administration (Gillon et al., 2021) and leads to food-drug interactions. The bioavailability of emodepside is reduced following oral administration in the fed versus fasting state (Gillon et al., 2020). The prolonged elimination half-life, which appears connected to enterohepatic recycling, increases the systemic exposure of microfilariae and can increase the macrofilaricidal effect. Optimization of the emodepside dose is important to achieve therapeutic concentrations and minimize toxicity. **Figure 2B** summarizes the pharmacokinetic pathway for emodepside.

## Efficacy and safety

The efficacy and safety of emodepside has been demonstrated against nematode parasites of cats and dogs (Reinemeyer et al., 2005; Schimmel et al., 2011; Altreuther et al., 2011; Böhm et al., 2015; Lee et al., 2019). Emodepside’s potent activity against microfilaria and adult filarial nematodes makes it a potential treatment for neglected tropical infections, including human onchocerciasis (Hübner et al., 2021; Kashyap et al., 2019; Bah et al., 2021). Human Phase IIa trials using dose-ranging, randomized, controlled studies have described the efficacy of emodepside against *Trichuris trichiura* and hookworm infections (Mrimi et al., 2023).

Repeated dose studies in humans demonstrated emodepside’s safety up to 40 mg per participant with good tolerance up to 20 mg (Gillon et al., 2018). The main adverse effect, visual disturbances, observed in Phase I clinical trials in healthy male subjects were mild and transient (Gillon et al., 2021). Neurotoxicity has occasionally been reported in dogs with a homozygous mutation in the MDR1 gene that codes for a P-glycoprotein (Noack et al., 2021). Symptoms include vomiting, incoordination, seizures, muscle tremors, and dilated pupils, which are more likely to be seen in fed dogs because of the faster absorption from the GI tract (Gaens et al., 2019; EMA, 2008a). The MDR1 mutation is more common in herding breed dogs, including Shetland Sheepdog, Australian Shepherds, Collies, and White Swiss Shepherd (Gramer et al., 2011; Noack et al., 2021). Animals with the P-glycoprotein defect exhibit neurological symptoms when treated with emodepside (Dias, 2014; Elmshäuser et al., 2015; Gaens et al., 2019; Noack et al., 2021) Thus, the presence of MDR1 mutant effects highlights the importance of pharmacogenomic factors that affect emodepside’s safety. Nevertheless, preclinical studies across various animal models have yet to identify significant safety concerns (EMA, 2008b), and current findings support a favorable safety profile for emodepside.

## Toxicity

This extensive distributions of SLO-1K channels in host organisms raise questions about potential toxicity of emodepside. Despite the importance of these channels, few studies detail emodepside’s toxicity. The research available generally points to low toxicity in different laboratory animals and pets, varying with the administration routes (EMA, 2008a; Vercruysse and Claerebout, 2014). The major toxicity observed has been in cases of overdose, noncompliance with dosing, and mutations in the multidrug resistance efflux carrier (EMA, 2008a; Gaens et al., 2019). **Supplementary Table S1** summarizes the observed toxic effects of emodepside.

### Overdose effects

Symptoms of depressed neurological and respiratory function have been observed in overdosed cats. Repeated dose toxicity studies revealed various adverse effects, including ataxia and increased motility, in rats (EMA, 2008a). The depressed neurological effects may be associated with an increased opening of ‘BK’ potassium channels (Crisford et al., 2015). The liver, adrenal glands, pancreas, and reproductive system are also target organs of repeated doses of emodepside (EMA, 2008a). The No-Observed Effect Levels (NOEL) vary with the mode of application (EMA, 2008a) and fasting status of the patient. Administration to a fasting human patient increases the possibility of observing toxic effects because of the increased absorption rate from the intestine.

### Neurological toxicity

Emodepside is a substrate for a P-glycoprotein, the multidrug resistance protein 1 (MDR1) which plays a crucial role in the uptake, body distribution, and elimination of numerous drugs and is expressed in different organs, including the brain (**Figure 2C**) (Elmshäuser et al., 2015; EMA, 2008b). Animals with the P-glycoprotein defect exhibit neurological symptoms when treated with emodepside (Gaens et al., 2019; Elmshäuser et al., 2015; Dias, 2014). Ataxia also arises in cats when they lick the spot-on application site immediately after treatment (EMA, 2008a). These observations highlight the importance of considering pharmacogenomic and pharmacokinetic variations in assessing safety.

### Reproductive toxicity

There are indications that emodepside might have the potential to interfere with embryo-fetal development (Wright and Elsheikha, 2018). Studies in rats and rabbits showed an impact on reproductive performance, only at doses causing parental toxicity, with no primary effect on fertility (CTP, 2014). Embryotoxicity/teratogenicity revealed some adverse effects, encompassing maternal toxicity, fetotoxicity, fetal malformations, and various skeletal/visceral anomalies or deviations; however, no issues were reported with emodepside in pregnant cats (CTP, 2014; Olliaro et al., 2011). Continued surveillance is needed to address any potential reproductive toxicity.

### Additional facets of toxicity

There is limited data on carcinogenicity, mutagenicity, and endocrine toxicity for emodepside. *In vitro* and *in vivo* studies show no evidence of genotoxicity, skin or eye irritations (CTP, 2014). However, rare local toxicities like alopecia, pruritis, and inflammation have been reported in cats (EMA, 2008a). Rat studies have revealed hormone deregulation as a cause of observed developmental toxicity (CTP, 2014). More studies are required to confirm these aspects of toxicity.

Studies in humans with emodepside have revealed mild and self-resolving side effects such as headache, visual disorders and dizziness (Gillon et al., 2021; Mrimi et al., 2023). Despite the ubiquitous distribution of BK channels in the body (exemplified in **Figure 2D**), the recognized target site of emodepside, the adverse effects are predominantly seen in ocular tissues possibly due to the specific need for the eye’s high spatiotemporal precision. The rapid absorption of emodepside revealed by its pharmacokinetic profile suggests that emodepside activates BK channels in retinal cells and the trabecular meshwork (Tanimoto et al., 2012; Cuppoletti et al., 2012), disrupting sharp vision and causing blurring. The response to BK channel activation by emodepside in other tissues may show less sensitivity and hence little side effects due to their unique physiological requirements and activation threshold or even differences in isoforms. While major toxicity reports have not been observed, a 40mg dose of emodepside per participant led to increased reports of eye and central nervous system disorders in some studies (Assmus et al., 2022; Gillon et al., 2020). Also, mutations in the MDR1 gene, which codes for P-glycoprotein, could affect its function and may be linked to symptoms like headaches. Care is required when administering emodepside to individuals with P-glycoprotein impairment or deficiency.

### Risk factors

The main risk factors associated with adverse effects of emodepside are noncompliance and MDR1 mutation predisposition. Caution is advised during administration. Again, emodepside’s filaricidal effect on adult parasites could pose a notable adverse reaction in dogs with *Dirofilaria immitis* because of its location in the pulmonary arteries: rapid paralysis of these parasites in the dog host results in pulmonary embolism and release of *Wolbachia* antigens. Additionally, there is limited data on emodepside’s use in severely debilitated animals (EMA, 2011; 2008a); thus, its use should be considered carefully. Potential risk factors with emodepside akin to those of diethylcarbamazine warrant further study.

## Conclusion

This review examines the safety profile of emodepside, focusing on its toxicological aspects within anthelmintic therapy. Emodepside’s distinct mechanism of action differentiates it from other anthelmintics, making it a promising option for tackling multidrug-resistant infections. The drug’s demonstrated safety, pharmacokinetics, and tolerability enhance its credibility. Additionally, the review provides insights into potential risks associated with emodepside and toxicity concerns.
