# Supplementary material for: Emodepside: the anthelmintic’s mode of action and toxicity
Source: Front Parasitol. 2024 Dec 10;3:1508167. doi: 10.3389/fpara.2024.1508167 (PMC11732007; doi:10.3389/fpara.2024.1508167)
Supplement: Supplementary file 1 [file Table1.docx]

**Table 1:** Summary of the reports of toxicity of emodepside and symptoms observed (EMA 2008b; Mrimi et al. 2023; Gillon et al. 2021; Gaens et al. 2019; Elmshäuser et al. 2015).

| Toxicity | Organism | Symptoms |
| --- | --- | --- |
| Dose toxicity | Rats | -Relative increase in brain weight  -Decrease weight gain  -Reduction in absolute weight of testes  -Piloerection  -Ataxia  -Increase motility  -Discolored feces  -Increase production of feces and  -Hyperglycemia  -Polydipsia. |
| Neurotoxicity | Humans | -Blurred vision  -Dizziness  -Headache  -Central Nervous System disorders  -Photophobia |
|  | Dogs | -Generalized tremors  -Panting  -Agitation  -Added concern with MDR1 mutations |
|  | Cats | -Mild ataxia and tremor  -lethargy and anorexia  -vomiting and hypersalivation  -Added concern with MDR1 mutations |
|  | Mice | -Ataxia  -Hyperkinetic effects |
| Reproductive Toxicity | Rats | -Affects reproductive performance |
| Embryotoxicity/Teratogenicity | Rats and Rabbits | -Possible fetal malformations and skeletal/visceral anomalies |
| Endocrine Toxicity | Rats | -Decreased estradiol  -Decreased triiodothyronine  -Decreased insulin  -Decreased leptin  -Decreased prolactin  -Increased thyroid stimulating hormone (TSH)  -Increased glucagon level |
| Local Toxicity | Cats | -Alopecia  -Pruritis  -Inflammation |

**Embryotoxicity/fetotoxicity, including teratogenicity.**

Embryotoxicity/fetotoxicity effects have been seen at high doses of emodepside in rodents. Signs of systemic maternal toxicity can be seen at oral dose rates ≥ 6 mg/kg in rats. At 18 mg/kg the signs of maternal toxicity were marked and associated with adverse effects on fetal development. The no observable effect level (NOEL) for rats for maternal toxicity was 2 mg/kg and the NOEL for fetal developmental toxicity was 0.5 mg/kg. In rabbits, the effects although like the rat studies required higher doses. The NOEL for developmental toxicity in rabbit was 5 mg/kg.

Emodepside is administered topically (spot-on) in cats but orally in dogs. It is recognized that salivation, vomiting and tremor can be seen at 10 times the recommended dose in cats and 5 time the recommended dose in kittens. MDR1 transporter mutations increase the chances of seeing this toxicity. The toxicity of emodepside during pregnancy has been investigated using different dose regimens in cats and the Committee for Veterinary Medicinal Products (CVMP) was satisfied that a single time point administration of the emodepside during pregnancy is likely to be well tolerated in cats and that the risk of reproductive toxicity is low. There remains a risk of embryotoxic effects at high or multiple dosing, however. The CVMP comments: “Although the product was well tolerated by pregnant cats, studies performed in rats and rabbits suggest that emodepside may interfere with embryo-fetal development. Therefore, women of child-bearing potential should avoid contact with, or wear disposable gloves when administering the product.”
